# Mild to Moderate Functional Tricuspid Regurgitation: Retrospective Comparison of Surgical and Conservative Treatment

**DOI:** 10.4061/2010/143878

**Published:** 2010-08-02

**Authors:** Michal Šmíd, Jakub Čech, Richard Rokyta, Patrik Roučka, Tomáš Hájek

**Affiliations:** ^1^Department of Internal Medicine, University Hospital and Medical Faculty Pilsen, Charles University Prague, alej Svobody 80, 304 60 Pilsen, Czech Republic; ^2^Department of Cardiac Surgery, University Hospital Pilsen, Alej Svobody 80, 306 60 Pilsen, Czech Republic

## Abstract

*Background*. Unoperated severe tricuspid regurgitation (TR) leads to the right ventricle (RV) failure. We wanted to determine if there was near-term postoperative progression of noncorrected mild to moderate functional TR in patients who underwent mitral valve surgery for chronic significant mitral regurgitation (MR) and if RV size and function were affected. *Methods and Results*. We compared two groups of patients retrospectively. In the first group (TVA+, *n* = 45), tricuspid valve annuloplasty (TVA) had been performed in conjunction with either mitral valve replacement (MVR) or mitral valve repair (MVP). The second group (TVA−, *n* = 22) underwent MVP or MVR without TVA. TVA+ group revealed a significant decrease in TR and right ventricle diameter. In the TVA− group, 7 patients (32%) showed a significant progression, by one or more grades, of noncorrected TR together with dilatation and decreased ejection fraction of the right ventricle. *Conclusions*. Tricuspid annuloplasty performed concurrently with MVP or MVR can prevent subsequent progression of tricuspid regurgitation along with right ventricular dilatation and systolic dysfunction in the near-term postoperative period.

## 1. Introduction

 There are two types of tricuspid regurgitation. Primary TR, attributed to congenital anomalies or resulting from bacterial endocarditis, is much less common than secondary (functional) TR [1]. Secondary TR is attributed to dilatation of the right ventricle and tricuspid annulus due to volume or pressure overloading of the right ventricle. 

The most frequent causes of functional TR are [2–5]

1. left heart disease (significant aortic or mitral valve disorder, or left ventricular dysfunction),
2. chronic pulmonary disease, and
3. primary pulmonary hypertension.

Reversible postcapillary or mixed pulmonary hypertension enabling surgery on an insufficient tricuspid valve usually accompanies significant chronic mitral regurgitation [6].

Functional TR may decrease or totally disappear after resolution of the left heart lesion responsible for the overloading of the right ventricle. However, TR progression occurs in as many as one half of patients [7, 8]. This untreated TR along with tricuspid annulus dilatation can lead to irreversible right ventricular dysfunction and failure [3].

When a separate tricuspid valve repair, due to significant TR, follows mitral valve surgery, mortality rates up to 32% are seen- and 5-year survivability is less than 50% [9, 10]. The reason is the poorer preoperative condition of the patients due to increased age, complications related to the previous mitral valve operation, and the possibility that irreversible right ventricular dysfunction had developed by the time of the second surgery.

**Figure 1:**
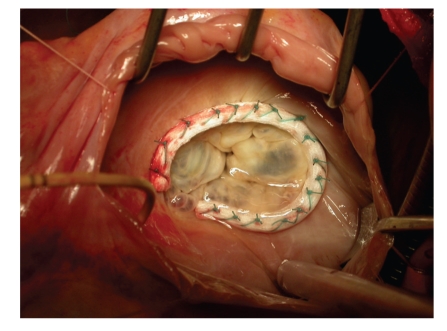
Tricuspid valve annuloplasty using semiflexible ring (provided Doc. Petr Němec, M.D., PhD., Center of Cardiovascular and Transplant Surgery, Brno, Czech Republic).

Because of the high total mortality following tricuspid valve replacement [5], valve repair is preferable [11], and if tricuspid valve replacement is indicated, a bioprosthetic valve is preferable to a mechanical one. Regarding the surgery-sparing techniques (for secondary dilatation of the tricuspid valve annulus with subsequent noncoaptation of the leaflets), placement of sutures around the circumference of the annulus was initially used to narrow the annulus (most frequently the surgery technique according to De Vega), but currently not only the narrowing but also the remodeling of the tricuspid annulus using annuloplasty ring is preferred (see Figure 1). The advantage of this procedure is a better long-term outcome of the sparing surgery.

Pulmonary hypertension, higher RV diameter with tricuspid valve annulus dilatation, and decreased RV ejection fraction are considered risk factors for deterioration of untreated tricuspid regurgitation following mitral valve surgery [7, 12]. Therefore tricuspid valve repair in conjunction with mitral valve surgery is beneficial for severe TR and should be considered for less than severe TR when there is dilated annulus (>40 mm) or pulmonary hypertension [7, 11, 13].

The objective of this retrospective analysis was to compare the development of untreated mildly to moderately significant functional TR after an operation for chronic severe mitral regurgitation in the near-term postoperative period between a group of patients who had MVR or MVP only and a group of patients who had both (MVR or MVP) and TVA simultaneously.

## 2. Patients and Methods

 We performed a retrospective analysis of 45 patients (TVA+ group) who underwent repair or replacement of the mitral valve due to significant chronic mitral regurgitation of ischemic or degenerative etiology. Simultaneously, tricuspid valve annuloplasty was performed with an annuloplastic ring if the patient had an annulus dilatation greater than 40 mm and if at least trace TR was present. This group of patients was compared with 22 patients (TVA− group) who underwent only repair or replacement of the mitral valve. While these patients' also had an annulus dilated with more than 40 mm and had at least trace TR, TVA was not performed. Patients with structural tricuspid valve disorder were not included in the study. There were no significant differences between the two groups of patients in terms of age, initial left and right ventricle ejection fraction, initial tricuspid and mitral regurgitation, and right ventricle diameter. Estimation of functional stage, using NYHA classification (New York Heart Association), was done through patients' questioning. The patients in TVA+ group were in a significantly higher NYHA class than patients in the TVA− group (Table 1). 

### 2.1. Echocardiography

 A transthoracic echocardiographic examination was performed on both groups of patients before the operative procedure and again 3 months following the procedure. The ejection fraction of both ventricles was assessed; the right ventricle diameter in long-axis parasternal view (PLAX) was measured. TR grade was assessed semiquantitatively according to Color Doppler Flow (CFM) from the apical four-chamber view (0,5 degree: trace, First degree: to 1/3 of the right atrium (RA), Second degree: 1/3–1/2 of RA, Third degree: 1/2–2/3 of RA, Forth degree: 2/3–the full length of RA). Mitral regurgitation was also assessed semiquantitatively using CFM from the apical four-chamber view. The estimation of systolic pressure in the pulmonary artery, based on the peak regurgitation gradient of the tricuspid valve, was not performed for all patients. Therefore, this value was not included in the retrospective analysis. 

### 2.2. Surgical Technique

 All patients were operated on via median sternotomy. Crystalloid antegrade cardioplegia was used as myocardial protection. In the TVA+ group, mitral valve repair was performed on 35 patients (78%) and mitral valve replacement on 10 patients (22%), while 22 patients (49%) also underwent aortocoronary bypass graft (CABG). Additionally, 19 patients (42%) underwent the MAZE procedure. In the TVA− group, mitral valve repair was performed on 16 patients (73%), valve replacement on 6 patients (27%), while 15 patients (68%) underwent CABG. Additionally, 7 patients (32%) underwent the MAZE procedure. In both groups, CABG was performed either as a primary indication or as a supplementary operation. The survey is shown in Table 2.

### 2.3. Statistical Methods

 The Student's **t**-test and Mann-Whitney U test were used for statistical evaluation.

## 3. Results

**Figure 2:**
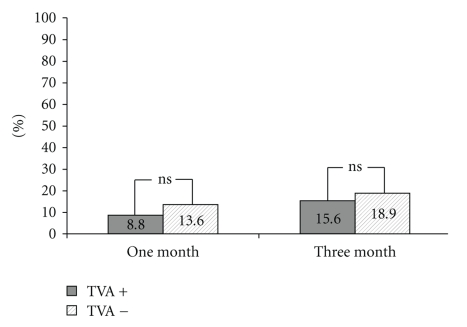
One month and three months mortality in TVA+ and TVA− groups.

 There was a trend for a higher one-month and three-month mortality in TVA− group (Figure 2). The NYHA class improved for both groups. The TVA+ group showed a statistically significant decrease in right ventricle diameter but a nonsignificant increase in right and left ventricle ejection fractions. The decrease in the average grade of TR was statistically significant (Table 3). None of the patients in the TVA+ group experienced progression in TR by more than one grade. 

 Like the TVA+ group, the TVA− group showed a statistically significant NYHA class improvement as well as a TR-grade decrease and a non-significant increase in the ejection fraction of both ventricles. However, the TVA− group showed a statistically significant dilatation of the right ventricle (Table 4). Seven patients (32%), from the TVA− group had postoperative TR progression by more than one grade with clinically significant right ventricular dilatation and decreases in ejection fraction (Table 5). There were no differences in the baseline characteristics (age, NYHA class, echocardiographic parameters) between these seven patients and the rest of the TVA- group.

## 4. Discussion

 The significance of TR has been often overlooked in cardiosurgery [8], however the importance of this issue has been recently addressed in the updated guidelines of both the ACC/AHA and ESC valve disease [11, 13]. 

 Most patients in the TVA+ group showed a significant decrease in TR grade, and there were no instances of annuloplasty failure reported. The reason that 1/3 of the patients showed no significant decrease in TR after TVA is due to the fact that in these patients only trace TR was present at the time of the operation, and trace TR remained even after the operation. A significant improvement in dyspnea was obvious in both groups, and it can be attributed to the resolution of the left heart lesion or, in selected cases, lesion resolution plus revascularization. Left ventricular function was not significantly affected by the mitral valve operation in either group. 

 The TVA− group also showed a statistically significant decrease in TR. We can only speculate that the most probable cause was the that resolution of the left heart lesion brought about a subsequent reduction in pulmonary hypertension, however pulmonary hypertension was not systematically measured in our study. If this speculation is correct, then it would, at least in the near-term postoperative period, oppose the statement that pulmonary hypertension is not a significant determinant of functional TR [8, 14, 15]. About 1/3 of the patients in the TVA− group showed TR progression by more than one grade and entered the classification of moderate to severe TR combined with dilatation and decreased right ventricle function. The percentage of patients experiencing a progression in TR is in agreement with the data presented in the literature [7, 8]. Nevertheless, the follow-up periods in these studies were markedly longer, ranging from one to ten years [7, 8, 15]. In the study by Metsunaga and Duran [8], the number of patients with functional TR after mitral valve repair gradually increased with time, and so we cannot exclude that in a longer follow-up; the percentage of patients who experienced a progression of TR would also be higher in our study. A significant progression in TR appeared only in some of the patients in the TVA− group. However, none of the patients in the TVA+ group experienced progression in TR by more than one grade, which supports the hypothesis that noncorrected preoperative tricuspid annulus dilatation can lead to postoperative TR progression [7]. Another reason for TR progression can be a temporary increase in pulmonary vascular resistance following surgery, which is related to extracorporeal circulation [16], as well as the possible influence of temporary postoperative hypervolemia.

## 5. Study limitations

 Our study has several limitations. First, it was retrospective in nature and non-randomized. Second, echocardiographic evaluation was performed only using transthoracic examination, and the evaluation of valve regurgitation was only semi-quantitative, which is in accordance with current clinical practice. Finally, the presence as well as the grade of pulmonary hypertension was not routinely echocardiographically assessed in all patients, so these data could not be used in our analysis.

## 6. Conclusion

 The results of our retrospective analysis support the latest guidelines for treatment of TR associated with mitral valve disease [11, 13]. These guidelines suggest, and our study substantiates, that concurrent tricuspid valve surgery should be considered for less than severe TR when there is dilated annulus >40 mm or pulmonary hypertension.

 We conclude that tricuspid valve annuloplasty of the tricuspid annulus dilated >40 mm, together with trace to moderate tricuspid regurgitation, performed concurrently with a mitral valve operation can prevent subsequent progression of tricuspid regurgitation and right ventricular dilatation and systolic dysfunction in the relatively near-term postoperative period.

## Figures and Tables

**Table 1:** Basic characteristics of study groups.

Parameter	TVA+	TVA−	*P*
*N*	45	22	
Men/women (*n*)	18/27	17/5	<.05
Age (years)	71.3 ± 6.8	70 ± 7.2	ns
NYHA	2.9 ± 0.6	2.6 ± 0.8	<.05
LV EF (%)	46.9 ± 13.5	41.1 ± 16.0	ns
RV EF (%)	46.2 ± 7.9	45.7 ± 8.2	ns
RV diameter (mm)	28.9 ± 4.0	28.8 ± 6.8	ns
TR grade	2.0 ± 0.8	1.7 ± 0.7	ns
MR grade	3.1 ± 0.5	3.2 ± 0.4	ns

TVA+ tricuspid valve annuloplasty, TVA− no tricuspid valve annuloplasty, NYHA: New York Heart Association classification, LV EF: left ventricle ejection fraction, RV EF: right ventricle ejection fraction, TR: tricuspid regurgitation, and MR: mitral regurgitation

**Table 2:** Performed operations.

Parameter	TVA+	TVA−	*P*
MVP (*n*)	35	16	ns
MVR (*n*)	10	6	ns
CABG (*n*)	22	15	ns
MAZE (*n*)	19	7	ns

TVA+ tricuspid annuloplasty, TVA− no tricuspid valve annuloplasty, CABG: coronary artery bypass graft, MVR: mitral valve replacement, and MVP: mitral valve repair

**Table 3:** Comparison of preoperative and 3-month-postoperative findings in patients with TVA+.

Parameter	Preoperative	Postoperative	*P*
NYHA	2.9 ± 0.6	1.6 ± 0.6	<.001
LV EF (%)	46.9 ± 13.5	47.3 ± 10.8	ns
RV EF (%)	46.2 ± 7.9	46.8 ± 7.2	ns
RV diameter (mm)	28.9 ± 4.0	26.5 ± 3.3	<.05
TR grade	2.0 ± 0.77	0.6 ± 0.5	<.001
MR grade	3.1 ± 0.5	0.5 ± 0.8	<.001

NYHA: New York Heart Association classification, LV EF: left ventricle ejection fraction, RV EF: right ventricle ejection fraction, TR: tricuspid regurgitation, and MR: mitral regurgitation.

**Table 4:** Comparison of preoperative and 3-month-postoperative values in patients with TVA−.

Parameter	Preoperative	Postoperative	*P*
NYHA	2.5 ± 0.8	1.5 ± 0.5	<.001
LV EF (%)	41.1 ± 16	41.3 ± 14.9	ns
RV EF (%)	45.7 ± 8.2	47.1 ± 5.7	ns
RV diameter (mm)	28.8 ± 6.0	32.3 ± 3.9	<.05
TR grade	1.7 ± 0.7	1.1 ± 1.2	<.05
MR grade	3.2 ± 0.4	0.2 ± 0.4	<.001

NYHA: New York Heart Association classification, LV EF: left ventricle ejection fraction, RV EF: right ventricle ejection fraction, TR: tricuspid regurgitation, and MR: mitral regurgitation.

**Table 5:** Comparison of preoperative and 3-month-postoperative findings in patients with TVA− and with TR progression by one or more grades.

Parameter	Preoperative	Postoperative	*P*
NYHA	2.4 ± 0.6	1.6 ± 0.5	<.05
LV EF (%)	47.5 ± 16.3	46.8 ± 17.6	ns
RV EF (%)	50.0 ± 0	43.3 ± 7.7	<.05
RV diameter (mm)	27.4 ± 3.4	34 ± 3.9	<.01
TR grade	1.1 ± 0.8	2.6 ± 0.5	<.001
MR grade	3.2 ± 0.5	0.5 ± 0.4	<.001

NYHA: New York Heart Association classification, LV EF: left ventricle ejection fraction, RV EF: right ventricle ejection fraction, TR: tricuspid regurgitation, and MR: mitral regurgitation.

## References

[1] Porter A, Shapira Y, Wurzel M (1999). Tricuspid regurgitation late after mitral valve replacement: clinical and echocardiographic evaluation. *Journal of Heart Valve Disease*.

[2] Sugimoto T, Okada M, Ozaki N, Hatakeyama T, Kawahira T (1999). Long-term evaluation of treatment for functional tricuspid regurgitation with regurgitant volume: characteristic differences based on primary cardiac lesion. *Journal of Thoracic and Cardiovascular Surgery*.

[3] Scully HE, Armstrong CS (1995). Tricuspid valve replacement: fifteen years of experience with mechanical prostheses and bioprostheses. *Journal of Thoracic and Cardiovascular Surgery*.

[4] Filsoufi F, Anyanwu AC, Salzberg SP, Frankel T, Cohn LH, Adams DH (2005). Long-term outcomes of tricuspid valve replacement in the current era. *Annals of Thoracic Surgery*.

[5] Rizzoli G, Vendramin I, Nesseris G, Bottio T, Guglielmi C, Schiavon L (2004). Biological or mechanical prostheses in tricuspid position? A meta-analysis of intra-institutional results. *Annals of Thoracic Surgery*.

[6] Widimský J, Widimský P (2003). *Základy monitorování hemodynamiky*.

[7] Dreyfus GD, Corbi PJ, Chan KMJ, Bahrami T (2005). Secondary tricuspid regurgitation or dilatation: which should be the criteria for surgical repair?. *Annals of Thoracic Surgery*.

[8] Matsunaga A, Duran CMG (2005). Progression of tricuspid regurgitation after repaired functional ischemic mitral regurgitation. *Circulation*.

[9] McCarthy PM, Bhudia SK, Rajeswaran J (2004). Tricuspid valve repair: durability and risk factors for failure. *Journal of Thoracic and Cardiovascular Surgery*.

[10] Van Nooten GJ, Caes F, Taeymans Y (1995). Tricuspid valve replacement: postoperative and long-term results. *Journal of Thoracic and Cardiovascular Surgery*.

[11] Vahanian A, Baumgartner H, Bax J (2007). Guidelines on the management of valvular heart disease: the task force on the management of valvular heart disease of the European society of cardiology. *European Heart Journal*.

[12] Colombo T, Russo C, Ciliberto GR (2001). Tricuspid regurgitation secondary to mitral valve disease: tricuspid annulus function as guide to tricuspid valve repair. *Cardiovascular Surgery*.

[13] Bonow RO, Carabello BA, Chatterjee K (2006). ACC/AHA 2006 guidelines for the management of patiens with valvular heart disease. *Journal of the American College of Cardiology*.

[14] Sagie A, Schwammenthal E, Padial LR, Vazquez de Prada JA, Weyman AE, Levine RA (1994). Determinants of functional tricuspid regurgitation in incomplete tricuspid valve closure: doppler color flow study of 109 patients. *Journal of the American College of Cardiology*.

[15] Matsuyama K, Matsumoto M, Sugita T, Nishizawa J, Tokuda Y, Matsuo T (2003). Predictors of residual tricuspid regurgitation after mitral valve surgery. *Annals of Thoracic Surgery*.

[16] Morita K, Ihnken K, Buckberg GD, Sherman MP, Ignarro LJ (1996). Pulmonary vasoconstriction due to impaired nitric oxide production after cardiopulmonary bypass. *Annals of Thoracic Surgery*.

